# Integrating microbial and host transcriptomics to characterize asthma-associated microbial communities

**DOI:** 10.1186/s12920-015-0121-1

**Published:** 2015-08-16

**Authors:** Eduardo Castro-Nallar, Ying Shen, Robert J. Freishtat, Marcos Pérez-Losada, Solaiappan Manimaran, Gang Liu, W. Evan Johnson, Keith A. Crandall

**Affiliations:** Computational Biology Institute, George Washington University, Ashburn, VA 20147 USA; Division of Computational Biomedicine, Boston University School of Medicine, Boston, MA 02118 USA; Division of Emergency Medicine, Children’s National Medical Center, Washington, DC 20010 USA; CIBIO-InBIO, Centro de Investigação em Biodiversidade e Recursos Genéticos, Universidade do Porto, Campus Agrário de Vairão, Vairão, 4485-661 Portugal; Universidad Andrés Bello, Center for Bioinformatics and Integrative Biology, Facultad de Ciencias Biológicas, Av. República 239, Santiago, 8370146 Chile

## Abstract

**Background:**

The relationships between infections in early life and asthma are not completely understood. Likewise, the clinical relevance of microbial communities present in the respiratory tract is only partially known. A number of microbiome studies analyzing respiratory tract samples have found increased proportions of gamma-Proteobacteria including *Haemophilus influenzae*, *Moraxella catarrhalis*, and Firmicutes such as *Streptococcus pneumoniae*. The aim of this study was to present a new approach that combines RNA microbial identification with host gene expression to characterize and validate metagenomic taxonomic profiling in individuals with asthma.

**Methods:**

Using whole metagenomic shotgun RNA sequencing, we characterized and compared the microbial communities of individuals, children and adolescents, with asthma and controls. The resulting data were analyzed by partitioning human and microbial reads. Microbial reads were then used to characterize the microbial diversity of each patient, and potential differences between asthmatic and healthy groups. Human reads were used to assess the expression of known genes involved in the host immune response to specific pathogens and detect potential differences between those with asthma and controls.

**Results:**

Microbial communities in the nasal cavities of children differed significantly between asthmatics and controls. After read count normalization, some bacterial species were significantly overrepresented in asthma patients (Wald test, p-value < 0.05), including *Escherichia coli* and *Psychrobacter*. Among these, *Moraxella catarrhalis* exhibited ~14-fold over abundance in asthmatics versus controls. Differential host gene expression analysis confirms that the presence of *Moraxella catarrhalis* is associated to a specific *M. catarrhalis* core gene signature expressed by the host.

**Conclusions:**

For the first time, we show the power of combining RNA taxonomic profiling and host gene expression signatures for microbial identification. Our approach not only identifies microbes from metagenomic data, but also adds support to these inferences by determining if the host is mounting a response against specific infectious agents. In particular, we show that *M. catarrhalis* is abundant in asthma patients but not in controls, and that its presence is associated with a specific host gene expression signature.

**Electronic supplementary material:**

The online version of this article (doi:10.1186/s12920-015-0121-1) contains supplementary material, which is available to authorized users.

## Background

The human microbiome [1] plays a key role in a variety of human health issues from obesity [2] to respiratory disease [3]. As we advance our understanding of the diversity of microbiomes across geography, time, individuals, and tissues within individuals, we become better positioned to take advantage of this growing wealth of information on the diversity of the human microbiome and how that diversity changes with infection and disease. Early studies capitalized on 16S ribosomal data for bacterial characterizations because of the ease of data collection and the robust and growing reference databases. However, with the declining costs of high-throughput sequencing (HTS) and the limitations of single gene inferences, microbiome studies are increasingly relying on shotgun metagenomics to obtain more complete profiles of microbial communities. An immediate concern is the sheer volume of data generated by the metagenomics approach, which presents novel challenges for efficient data handling and analysis. These challenges are especially acute when attempting to identify relevant microbes for suspected infections — trying to differentiate microbes relevant to the host from microbes that do not elicit a response from the host is a daunting task. A variety of techniques have been developed to isolate potential pathogens for HTS targets using molecular biological approaches. However, this limits the inferences with respect to the host response. Other approaches, for instance, dual RNA-Seq has been recently suggested as a promising approach to assess differential gene expression in both the pathogen and the host from the same sample [4].

Microbiome studies of human disease typically focus on correlates between microbial composition and disease phenotype at single or multiple points in time. However, this poses significant problems when it comes to elucidating potentially causal relationships. The lingering question is whether disease results in a certain microbiome or whether this microbiome is the underlying cause of the disease. Prospective studies have attempted to establish causality relationships by monitoring microbial populations before and after the onset of disease. In the case of asthma, prospective studies have identified *H. influenzae*, *M. catarrhalis*, and *S. pneumoniae* colonization as potential risks factors [5]. Colonization with these three bacterial species has also been linked to the development of severe pulmonary infections, however this association has only been seen in children that did not develop asthma [6]. In addition, the lung microbiome project and others have proposed a core pulmonary microbiome of healthy individuals that includes genera such as *Streptococcus*, *Haemophilus*, and *Pseudomonas* (same order as *M. catarrhalis*), which casts a shadow on elucidating the role of such bacterial species in asthma [7–9].

Here, we present a computational strategy – combining RNA microbial identification and host differential gene expression signatures – to identify pathogens associated with asthma in children and supported by differences in the patients’ responses to infection (host immune response-related gene expression signatures). We tested whether microbial composition (viral, fungal, and bacterial) is significantly different between asthma individuals and controls, and whether differentially abundant microbes with available host gene signatures are associated to genes related to the immune response by the host.

## Methods

### Sample collection

Participants were part of the AsthMaP (Asthma Severity Modifying Polymorphisms) Project (Table 1). The AsthMaP Project was a single-center observational study of asthma. AsthMaP participants ranged between the ages of 6 and 20 years, with physician-diagnosed asthma for at least one year prior to the time of recruitment from the emergency department, inpatient units and outpatient clinics. Individuals who reported a medical history of chronic or complex cardiorespiratory disease were ineligible. Control subjects were confirmed not to have asthma through negative response to multiple survey questions for asthma diagnosis, symptoms, medication use, and healthcare utilization. Specific AsthMaP methodology has been published elsewhere [10–13]. Our Institutional Review Board approved this study and parents and participants gave consent/assent.

**Table 1:** Demographic data from asthma and control subjects

Variable	Asthma (n = 8)	Control (n = 6)
	Mean (95 % CI)	Mean (95 % CI)
Gender, % male	75	83
Age, years, median (range)	11 (6, 17)	15 (10, 20)
FEV_1_ (% change with bronchodilator), median (range)	3.5 (−13, 10)	N/A
Post-bronchodilator FEV_1_ (% predicted), median (range)	97 (62, 107)	N/A
FEF_25–75_ (% predicted), median (range)	83 (28, 112)	N/A
Post-bronchodilator FEF_25–75_ (% predicted), median (range)	93 (37, 110)	N/A
Serum IgE, IU/mL, median (range)	247 (60, 1706)	N/A
Blood eosinophils, %, median (range)	6 (2, 14)	N/A
ACT score, median (range)	23 (17, 23)	N/A

*FEV*_*1*_ Forced Expiration Volume, *FEF* Forced Expiratory Flow, *ACT* Asthma Control Test. *N/A* = information not available

Nasal epithelial cells were collected from 8 children and adolescents with asthma and 6 healthy controls by brushing the medial aspect of the inferior turbinate of each nare using a cytology brush. Nasal samples are an accepted surrogate for bronchial samples [14] that have the advantage of being acquired using minimally invasive techniques. This sampling technique allows for ethical collection from the full range of asthma severity, including youth with mild asthma, and healthy controls.

Samples were collected from fresh tissues and macerated using sterilized plastic tips in 1.5 mL sterile tubes. Samples in Trizol were frozen immediately at −80 until a later date for batch RNA extraction and processing. Total RNA was extracted using Trizol reagent (Life Technologies) and the resulting lysate was used for affinity RNA purification in silica columns following manufacturer’s instructions (Norgen Biotek). RNA quality was assessed by measuring 260/280 absorbance ratio and by integrating proportions of RNA using microchip electrophoresis on Agilent Bioanalyzer 2100 RNA 6000 nanochips (Agilent, Palo Alto CA). Samples with an RNA Integrity Number value greater than five were used for subsequent analysis. Total RNA was subjected to RiboZero ribosomal RNA reduction prior to library preparation using Illumina TrueSeq Stranded Total RNA kit (San Diego, CA) and sequenced on a HiSeq 2500 instrument on two separate ‘Rapid’ flow cells. This generated an average of 41.4 million single-end 100 bp sequencing reads per sample. Sequence data was deposited in the Sequence Read Archive and can be found under the BioProject [SRA: PRJNA255523].

### Analyses

Reads were preprocessed using PRINSEQ-lite 0.20.4 and FastQC 0.10.1 (trimming reads and bases < 25 PHRED, removing exact duplicates, reads with undetermined bases, and low complexity reads using Dust filter = 30) [15]. We constructed a ‘target’ genome library containing all bacterial, fungal, and viral sequences from the Human Microbiome Project Reference Database (http://www.hmpdacc.org/reference_genomes/reference_genomes.php) using the PathoLib module from PathoScope 2.0 [16]. We aligned reads to these libraries using the Bowtie2 algorithm [17], and then filtered any reads that also aligned to the human genome (hg19) as implemented in PathoMap (−−very-sensitive-local -k 100 --score-min L,20,1.0). In these samples, an average of 1.8 million reads (9.1 %; range: 4.8 %-16.72 %) per sample aligned to the target libraries before filtering the human genome. We then applied PathoScope 2.0 – specifically the PathoID module – to characterize the microbial communities in each patient.

Exploratory analysis and differential species abundance testing were performed in R 3.1.2 and Bioconductor 3.0 [18, 19] using packages xlsx 0.5.7, gtools 3.4.1, CHNOSZ 1.0.3.1, plyr 1.8.1, ggplot2 1.0.0, reshape2 1.4.1, gplots 2.16.0, Phyloseq 1.10.0, and DESeq2 1.6.3 [20–28]. Briefly, various indices (Observed, Chao1, Shannon, Simpson) were obtained using the plot_richness function of the PhyloSeq package and beta diversity was obtained using the R base package [19, 26]. Numbers of mapped reads were normalized across all samples using the variance stabilizing transformation method [27, 28]. Relative differences between groups were tested using a Wald test (with Cook’s distance correction for outliers) and adjusted by applying the Benjamini-Hochberg method to correct for multiple hypotheses testing at alpha = 0.05 [29, 30]. Taxa whose base number of normalized reads was less than 50 were not considered. Principal coordinate analysis (PCoA) was performed on a Jensen-Shannon distance matrix derived from read counts aggregated by genus as estimated in PathoScope.

For gene differential expression analysis, we aligned the dataset to the human genome using TopHat v2.0.6 [31] and estimated the expressed gene abundance using Cufflinks v2.1.1 [32] represented as fragments per kilobase of exon per million fragments mapped (under default parameters). Since *M. catarrhalis* was detected with high proportion of mapped reads in 5/8 asthma samples while with low proportion of mapped reads in all of the control samples, we further evaluated the host response gene expression signature of *M. catarrhalis* in these samples. In a previous study, a list of differential expressed genes (77 genes) was identified in the respiratory tract epithelial cells in response to adherent *M. catarrhalis* BBH18 [33]. We applied an adaptive Bayesian factor analysis approach as implemented in the ASSIGN toolkit from Bioconductor [34]. We estimated the strength of *M. catarrhalis* host gene expression signature onto the samples in our dataset to determine whether this signature is present in the tissue samples of asthma and control samples [16]. For this analysis we used the following parameters: [adaptive_B = TRUE, adaptive_S = TRUE, mixture_beta = TRUE, p_beta = 0.001, iter = 2000, burn_in = 1000, theta0 = 0.05, theta1 = 0.9].

## Results and discussion

To our knowledge, this is the first study reporting the use of shotgun RNA sequencing for microbial identification and differential host gene expression. Throughout this study we refer to microbial composition as the combined effect of the presence of a certain microbe and its gene expression. The advantage of this strategy is that measures of relative abundance are related to the actual activity or expression of a microbe at a given point in time instead of to the census number of a microbe. Additionally, it allows for the interrogation of the host transcriptomes or specific gene signatures from the same sequence dataset. Franzosa et al. showed that metagenomic and metatranscriptomic genes and/or species abundance do not necessarily correlate [35], meaning that species’ relative abundances reported in this study represent actual activity or expression of microbes, and might not correlate to relative abundance from shotgun DNA experiments.

### Asthma microbial communities are less diverse than controls

**Fig. 1:**
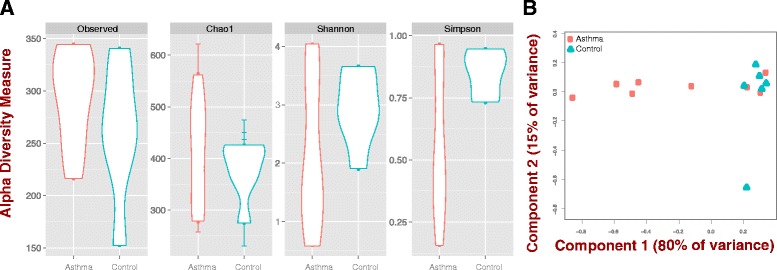
Alpha and beta diversity for asthma and control samples as estimated by different distance metrics. **a** Alpha diversity measures show controls are more diverse than asthma individuals in metrics that account for evenness, however in asthma individuals we observed more species. Observed = observed diversity; Chao1 = Chao estimator; Shannon = Shannon diversity index; Simpson = Simpson diversity index. **b** Multidimensional scaling using principal coordinate analysis (PCoA). Coordinates 1 and 2 explain 95 % of the observed variance

**Fig. 2:**
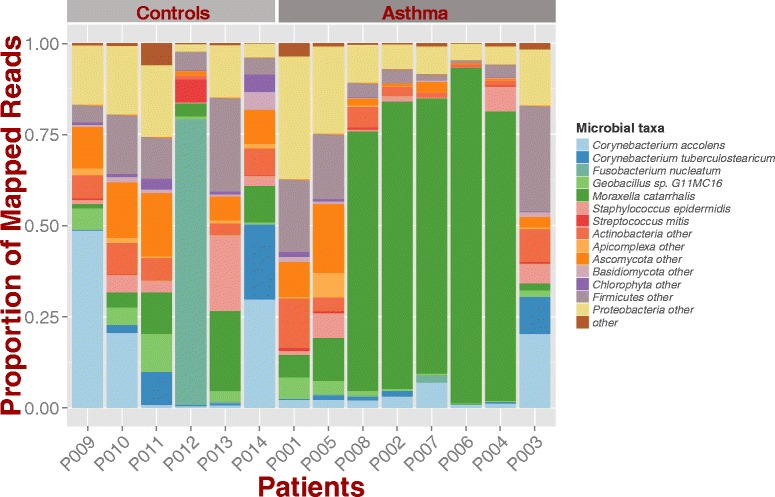
Microbial composition of asthma and control samples. Stacked bar chart shows different composition among groups with *Moraxella catarrhalis* dominating 5 out of 8 asthma samples. Since samples are RNA, the proportion of mapped reads represents the confounded variable of microbe presence and microbial gene expression

We performed analyses of alpha and beta diversity to assess species richness and evenness within and among samples (Fig. 1 and b). We obtained estimates of various indices to characterize the richness and heterogeneity of the samples partitioned by asthma and control samples (Observed, Chao1, Shannon, Simpson). Observed and Chao1 are measures of species richness (number of species); the latter including a correction for unobserved species [36, 37]. In turn, Shannon and Simpson incorporate relative species abundance and thus represent Evenness or Heterogeneity [38]. We observed that asthma samples have more species (richer) compared to control individuals (Fig. 1a; Observed and Chao1). However, measures that explicitly model Evenness (Shannon and Simpson indices) suggest that asthmatic samples are dominated by fewer species (5 of 8 cases dominated by *Moraxella catarrhalis*; Fig. 1a; Fig. 2) and are thus less diverse than controls.

Decreased microbial diversity have also been observed in other human diseases [39–41], although increased diversity in diseased patients has also been noted [42]. In asthma studies, bacterial diversity, as surveyed by 16S rRNA amplicon sequencing and 16S microarray typing, exhibits an opposite trend to bronchial and induced sputum samples, i.e., asthma samples are more diverse than controls [43, 44]. In addition, other studies have not detected significant differences among asthma samples and controls [9, 45]. The discordance between our results and previous studies might arise from two sources. We used shotgun RNA sequencing instead of marker-based approaches. The shotgun approach is more comprehensive (virus, bacteria, fungi); thus, it is likely that we sampled the microbiome more extensively. In addition, our estimates might not be directly comparable as we measure abundance as a composite of the product of microbial gene expression and census numbers, i.e., we sampled species that were expressing genes vs. sampling species that were present. Secondly, we sampled a surrogate of bronchial samples, i.e., the nasal cavity of children and adolescents (Table 1), and other studies have directly sampled the lower respiratory tract of adults and children.

Regarding among-sample relatedness, we observe that the five samples where *M. catarrhalis* is relatively more abundant tend to differentiate from controls (PCoA; 95 % of variance), while asthma samples with low levels of *M. catarrhalis* tend to cluster with controls (Fig. 1b). Interestingly, the latter three samples also exhibit the lowest proportion of *M. catarrhalis*, and two of them exhibit no host response to *M. catarrhalis*-associated genes (below; Fig. 4; P001 and P005). Recently, Goleva et al. found no differences either in diversity or composition in patients with corticoid-sensitive or resistant phenotypes compared to controls in samples without *M. catarrhalis* [45]. This suggests that asthma microbiomes where *M. catarrhalis* is not detected resemble those of control individuals, however we do not discard the possibility that another unidentified microbe is driving this apparent similarity.

### Microbial identification and relative abundance in asthma and control communities

The resulting composition differed significantly between the cases and controls at the species level, with 5 of the 8 cases showing high (more than 50 % of mapped reads) prevalence of the bacterial species *M. catarrhalis* (Fig. 2; check Additional File 1 for raw counts). Other abundant species in asthma samples were *Corynebacterium accolens*, and *C. tuberculostearicum*. However, these were also found in high abundance in control samples (Fig. 2). *Corynebacterium spp.* have been detected in sinus nasal studies of healthy individuals as well as in cases of rhinitis and rhinosinusitis, where their prevalence nears 100 % and their abundance is relatively high [46–48].

**Fig. 3:**
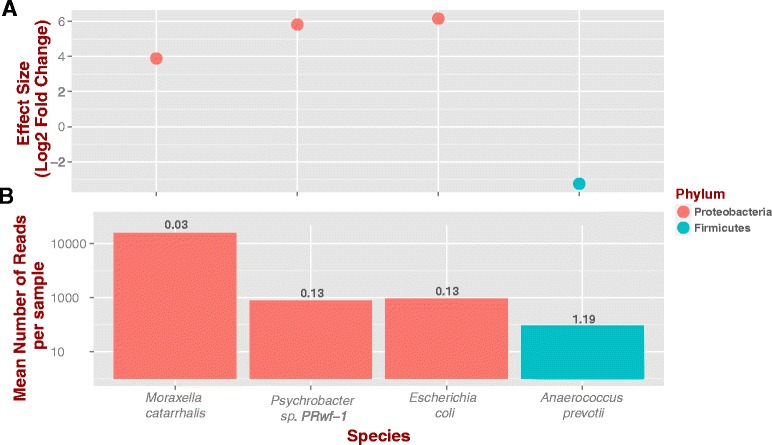
Effect size for asthma samples over controls (y axis) as a function of species (x axis), colored by phylum. **a** Effect size was computed by normalizing read counts and comparing asthma and control samples using a Wald test at α = 0.05. **b** On average *Moraxella catarrhalis* asthma samples exhibit more reads than the other species identified (y-axis is Log10). The number on top of bars represent the coefficient of variation (standard deviation/mean)

When we formally test for differential relative abundance, we observed a log_2_–based effect size of 3.8 for *M. catarrhalis*, i.e., this species is on average 14 times more abundant in children with asthma than controls (Fig. 3a). These findings build on previous metagenomic surveys using 16S rRNA, which found increased proportions of Proteobacteria in cases but not controls, speculating that this could be explained by *Moraxella spp*. and *Hemophilus spp.* [9]*. M. catarrhalis* is a pathogen associated with pneumonia in early childhood [49], and airway colonization shortly after birth with *M. catarrhalis*, along with *H. influenzae, and S. pneumonia*, is associated with later asthma development [5] and with wheezy episodes in young children [50].

We also detected *Escherichia* and *Psychrobacter* (Family: Moraxellaceae) to be significantly more abundant in asthma samples than controls, both members of the human microbiome [51, 52]; yet in low quantities (Fig. 3b; 65 and 55 reads, respectively). While we detected *H. influenzae, Streptococcus* spp., and *Staphylococcus* spp. in asthma samples, their abundance was not significantly different between asthma and control samples (p-value > 0.05; check Additional File 2 for R code). We also found *Anaerococcus prevotii*, member of the normal microbiome of the skin, oral cavity and the gut, to be relatively less abundant in asthma samples (Fig. 3a-b; 57 reads on average). These findings, i.e., more Proteobacteria and less Firmicutes in asthma, are in agreement with other reports [9, 44].

### Host gene expression validates microbial community profiling

**Fig. 4:**
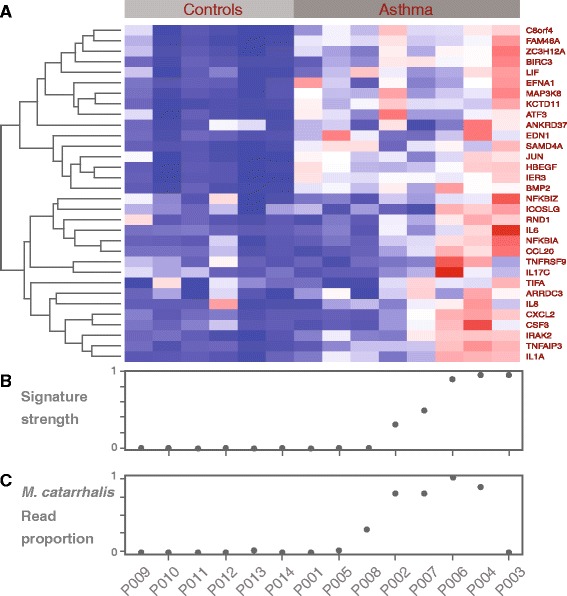
**a** Heatmap of *Moraxella catarrhalis* signature genes distinguishes the asthma samples from the controls. The color scale goes from blue (low expression) to red (high expression). **b**, **c** The *Moraxella catarrhalis* signature strengths are highly concordant with the PathoScope read proportions in control and asthma samples with the exception of sample P003

While identifying pathogenic species of a distinct airway microbiome in cases but not in controls is suggestive evidence to implicate an agent for disease, we wanted to further validate this conclusion by examining the host response to *M. catarrhalis*. Because our starting nucleic acid material is RNA from human epithelial cells, the majority of the sequencing reads are of human origin (>95 % mapping to human genome; ~75 % mapping to human transcriptome). Thus, we can capitalize on these data to examine host response gene expression through these transcriptomic data. We obtained a set of 77 genes that were previously associated with the immune response to *M. catarrhalis* infection in respiratory tract epithelial cells [33] of which 32 gene names were found in our dataset. We fit this *M. catarrhalis* host response gene expression signature onto our asthma and control samples (Fig. 4a). None of the controls expressed the *M. catarrhalis* response signature (Fig. 4b). For the eight asthma samples, five exhibited a high *M. catarrhalis* signature strength. These five samples included the samples with the four highest scoring read proportions from PathoScope. Samples with high proportion of *M. catarrhalis* exhibited increased expression of mediators of inflammation (e.g., CCL20; IL1A; IRAK2) and apoptosis (e.g., TNF; C8orf4; Fig. 4a).

Additionally, signature strength and read map proportions are in concordance for the majority (4 out of 5) of the patient samples (Fig. 4b, c). However, there was one discordant sample with a very low *M. catarrhalis* read proportion (0.02) that scored very high with respect to the gene expression signature (P003 in Fig. 4c). In this sample, we did identify 281 reads from *Moraxella spp*., indicating that we could still be observing a true host response. Alternatively, this could be a false positive due to lack of specificity in the signature. For instance, in sample P003, we also detected 5249 reads for *Corynebacterium*, representing ~0.3 proportion of mapped reads. We did not find a specific signature for *Corynebacterium* in the literature, however, species in this genus are known to trigger inflammation in the nasal cavity and sinuses, which might explain the strong pro-inflammatory response [46, 53]. Altogether, this illustrates the future need for developing a multi-signature approach (i.e., immune response caused by multiple pathogens) that can distinguish between related response signatures. The other two asthma samples (P001 and P005) with no detectable signature for *Moraxella* showed 304 and 1490 *M. catarrhalis* normalized reads (6 and 12 %, respectively). In agreement with our findings, Følsgaard et al. (2014) detected local inflammation markers in nasal mucosal lining fluid samples of neonates after colonization by *M. catarrhalis*, which might lead to the establishment of chronic inflammation [54].

## Conclusions

Our study demonstrates the efficacy of combining microbial identification and host gene signatures for microbial characterization under asymptomatic conditions. In a single shotgun RNA experiment, our integrative approach shows the dominating presence of *M. catarrhalis* in the airways of asthmatic children and the strength of the host immune response against it. This suggests that the airways of asthmatics are chronically inflamed, which may be associated with their ability to respond against opportunistic infections.

While the small sample size of our study, the small number of gene signatures available, and the need for a multi-signature approach render our results as preliminary, we show that our approach simultaneously characterizes the diversity of microbial communities (bacteria, fungi and viruses), and the differential expression of loci from the host in response to an infection. Such a dual approach allows for robust diagnosis in human health and has a direct and broad applicability in epidemiological, ecological, and medical studies. Future development of multi-species signature statistical approaches along with the availability of more gene signatures will strengthen microbial detection by RNA microbial profiling and host differential gene expression.

## Additional files

Additional file 1:
**Spreadsheet with taxonomy of species detected and their abundances as read counts.** (XLSX 143 kb) Additional file 2:
**OTU and taxonomy tables, metadata, and R code for analysis of differential abundance.** (ZIP 32 kb)

## Abbreviations

rRNA: Ribosomal ribonucleic acid
RIN: RNA integrity number
HTS: High-throughput sequencing
SRA: Sequence read archive
